# Effects of single neuron firing patterns on network dynamics

**DOI:** 10.1186/1471-2202-14-S1-P227

**Published:** 2013-07-08

**Authors:** Ajith Padmanabhan, Ioannis Vlachos, Ad Aertsen, Arvind Kumar

**Affiliations:** 1 Neurobiology & Biophysics, Faculty of Biology, University of Freiburg, Freiburg, Germany; 2 Bernstein Center Freiburg, Freiburg, Germany

## 

The interaction of activities at multiple levels, ranging from molecular to cellular to network level, is a fundamental aspect of information processing in the brain. In vitro studies show that the firing patterns of neurons in response to an input current are highly diverse. Recent experimental data suggest that parvalbumin and somatostatin expressing interneurons, differing in their connectivity and firing patterns, influence the orientation selectivity of pyramidal neurons differently. However, under which conditions low-level neuron properties like spiking patterns affect the network activity dynamics remains to be understood. Therefore, we studied the dynamics of spiking neuronal networks (SNN) by systematically varying the firing pattern of inhibitory interneurons from fast spiking to bursting, keeping the excitatory population as regular spiking.

In an SNN with sparse and homogeneous connectivity, global network properties such as population synchrony and mean firing rates did not show significant differences with variations in the type of inhibitory neurons. Interestingly, local properties such as burstiness of the individual neurons were determined by the global network state for instance in the asynchronous activity state both fast spiking neurons and bursting neurons exhibited similar spiking patterns. Thus, the global network state, instead of the neurons' intrinsic properties, determined the spiking pattern of the neurons.

Because the effect of neuronal spike patterns could be obscured by the random and homogeneous connectivity, we considered two specific deviations: First we introduced hubs into the network by allowing the fast spiking and bursting neurons to form up to eight times more connections. The overall out degree in such a network with hubs was kept the same as in the random homogeneous networks. Such topology did not affect the network dynamics qualitatively.

Next, we separated the inhibitory population into two sub-populations, connected in feedforward (FF) and feedback (FB) manner. With this connectivity scheme, we initially found that neuronal spike patterns can affect the oscillation frequency of the population activity if the fast spiking inhibitory neurons were present as the FB population and FF population was altered between the various ratios of fast spiking and bursting neurons. However, the differences in the oscillations frequency could be attributed to the different gain of the two types of neurons. That is, it was an effect of differences in the degree of recurrent inhibition and not that of spiking activity patterns.

In summary, our results suggest that for random homogeneous and the two types of inhomogeneous recurrent SNNs, the individual neuronal patterns do not affect the global dynamics. Any differences in global dynamics with change in single neuron firing patterns is due to the ensuing difference in the firing rate of the neuron types. On the other hand, the global activity state influenced the local parameters like burstiness.

